# Comparative Effectiveness of Acupuncture and Antiarrhythmic Drugs for the Prevention of Cardiac Arrhythmias: A Systematic Review and Meta-analysis of Randomized Controlled Trials

**DOI:** 10.3389/fphys.2017.00358

**Published:** 2017-06-08

**Authors:** Yanda Li, Hector Barajas-Martinez, Bo Li, Yonghong Gao, Zhenpeng Zhang, Hongcai Shang, Yanwei Xing, Dan Hu

**Affiliations:** ^1^Guang'anmen Hospital, Chinese Academy of Chinese Medical SciencesBeijing, China; ^2^Masonic Medical Research LaboratoryNew York, NY, United States; ^3^Xi Yuan Hospital, Chinese Academy of Chinese Medical SciencesBeijing, China; ^4^Key Laboratory of Chinese Internal Medicine of the Ministry of Education, Dongzhimen Hospital Affiliated to Beijing University of Chinese MedicineBeijing, China; ^5^Department of Cardiology and Cardiovascular Research Institute, Renmin Hospital of Wuhan UniversityWuhan, China

**Keywords:** arrhythmias, acupuncture, meta-analysis, antiarrhythmic drug, randomized controlled trials

## Abstract

**Introduction and Objectives:** This study was designed to systematically evaluate the effectiveness of acupuncture treatment for arrhythmia compared to existing drug therapy.

**Methods:** Randomized controlled trials (RCTs) were identified through searches of the MEDLINE, CNKI, Embase, and Cochrane databases (1970 through 2016) and hand searches of cross-references from original articles and reviews. Clinical trials that randomized arrhythmia patients to acupuncture therapy vs. conventional drugs, sham acupuncture, or bed rest were included for analysis.

**Results:** A total of 13 trials with 797 patients met the criteria for analysis. The results of the meta-analysis showed no statistically significant difference between acupuncture and conventional treatment for paroxysmal supraventricular tachycardia (PSVT) (*n* = 203; RR, 1.18; 95% CI 0.78–1.79; *I*^2^ = 80%; *P* = 0.44). However, in the ventricular premature beat (VPB) group, it showed a significant benefit of acupuncture plus oral administration of anti-arrhythmic drug (AAD) on response rates compared with the oral administration of AAD (*n* = 286; RR, 1.15; 95% CI 1.05–1.27; *I*^2^ = 0%; *P* = 0.002). Finally, when compared with the sinus tachycardia (ST) cases without any treatment, acupuncture has benefited these patients (*n* = 120; MD, 18.80, 95% CI 12.68–24.92; *I*^2^ = 81%; *P* < 0.00001).

**Conclusions:** In summary, our meta-analysis demonstrates that clinical efficacy of acupuncture is not less than AAD for PSVT. Furthermore, in sub-group analysis, acupuncture with or without AAD, shows a clear benefit in treating VPB and ST. However, more definitive RCTs are warranted to guide clinical practice.

## Introduction

The 2013 overall rate of death attributable to cardiovascular diseases was 222.9 per 100,000 Americans. Among cardiovascular diseases, cardiac arrhythmia is one of the most common and serious diseases and is a serious threat to human health (Chen et al., 2013; Mozaffarian et al., 2016). Arrhythmia mainly refers to the abnormal of heart impulse frequency, rhythm, origin site, conduction velocity, or the excited order. Arrhythmias include: paroxysmal supraventricular tachycardia (PSVT), sinus tachycardia (ST), ventricular premature beat (VPB), atrial fibrillation (Af), etc., (Burashnikov et al., 2012; Mozaffarian et al., 2016).

Commonly used clinical treatments for arrhythmia include drug therapy, surgical intervention, and radiofrequency catheter ablation. However, all antiarrhythmic drugs have proarrhythmic effects and may cause gastrointestinal reactions, central response, hypotension, and other adverse reactions (Camm, 2017). Surgical treatment may cause serious damage and is always complex. Radiofrequency catheter ablation is characterized by a low recurrence rate and fewer complications, but it has an unsatisfactory success rate (Lomuscio et al., 2011).

In recent years, a number of clinical observations indicate that acupuncture might be an effective therapy for cardiac arrhythmias through multiple mechanisms and has the advantages of being simple, safe, inexpensive, and associated with few adverse reactions (Lomuscio et al., 2011). Recent scientific studies have examined the role that acupuncture may have as an effective intervention for cardiac arrhythmias (Kim et al., 2011; Lin, 2015). Although plagued by methodological shortcomings, these studies support acupuncture as an effective treatment for atrial flutter, (Xu and Zhang, 2007; Lomuscio et al., 2011) PSVT (Wu and Lin, 2006) inappropriate ST, (Xie et al., 2004) and symptomatic PVBs (Yuan and Ai, 2002; Liu, 2005; Zhong, 2008). Traditional Chinese Medicine (TCM) believes that the normal heartbeat relies on the “heart-qi.” Ample “heart-qi” maintains normal cardiac rhythm (Han and Wang, 2008; Andrew and Mehmet, 2014).

This study was designed to systematically evaluate the use of acupuncture for arrhythmia treatments, and to evaluate the effectiveness of acupuncture treatment for arrhythmia compared to existing drug therapy.

## Methods

This systematic review was conducted in accordance with the Preferred Reporting Items for Systematic Reviews and Meta-Analyses guidelines. Ethical approval was not necessary for this review study.

### Search strategy

Studies were identified through a computerized literature search of MEDLINE, Embase, the Cochrane library and CNKI from their inception through January 2016. The search strategy terms used were as follows: (Acupuncture OR Acupuncture Therapy OR Therapy, Acupuncture) AND (Arrhythmias, Cardiac OR Cardiac Dysrhythmia OR Dysrhythmia, Cardiac OR Cardiac Arrhythmia OR Cardiac Arrhythmias OR Arrhythmia OR Arrhythmia) AND (randomized controlled trial OR controlled clinical trial OR randomized OR placebo OR clinical trials as topic OR randomly OR trial).

### Selection criteria

RCTs were included with blind method or not and without language restriction. Trials were included if they enrolled patients with various types of cardiac arrhythmia and randomly allocated patients to receive acupuncture treatment or commonly used clinical treatments. Studies were excluded if they meet any of the following conditions: (1) *Post-hoc* analysis or studies without reliable outcome data; (2) Non-randomized controlled trials; (3) Auricular needling or ear acupuncture were chosen.

### Observed outcome measures

Validity was calculated as follows: In the analysis evaluating the effectiveness of acupuncture for PSVT, Reduction of heart rate (HR) and restoration to normal sinus rhythm (NSR) were set as the outcome measures. In the analysis assessing the effectiveness of acupuncture for VPB, symptom reduction and HR reduction and % reduction of VBP on ECG or holter were set as the outcome measures. And in the analysis assessing the effect of a single acupuncture treatment in patients with ST, Reduction of HR was set as the outcome measure.

### Statistical analysis

Based on the different types of arrhythmia, we divided all of the studies into PSVT, VPB, and Af groups for assessment. The data were extracted from each of the published studies independently by two investigators. All *P*-values were two-tailed, with statistical significance set at 0.05, and the confidence intervals were calculated at the 95% level. Analyses were performed using Revman software version 5.3. Publication bias was detected to use funnel plots, with asymmetry suggesting possible publication bias. Publication bias was also assessed by Begg test, Egger test, and the funnel plots made using the STATA software version 12.0 for the meta-analysis. Publication bias was considered to exist if the *P*-value was <0.05.

## Results

### Baseline characteristics

**Figure 1:**
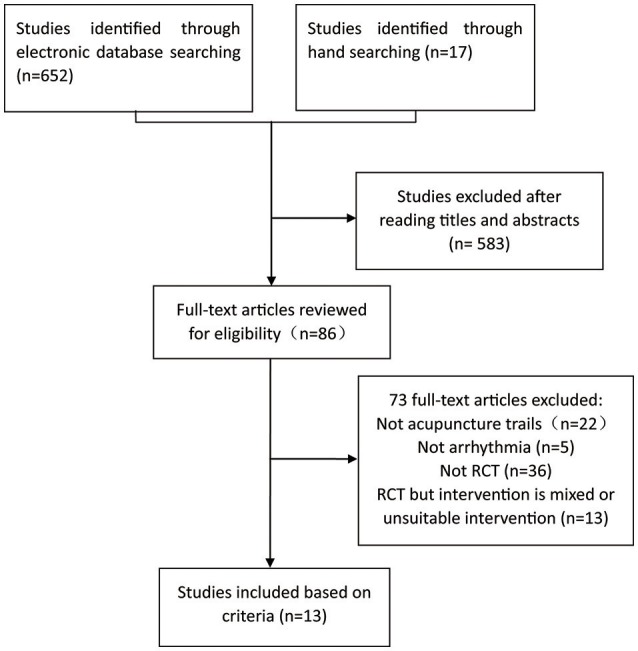
Flow diagram of the systematic review.

**Figure 2:**
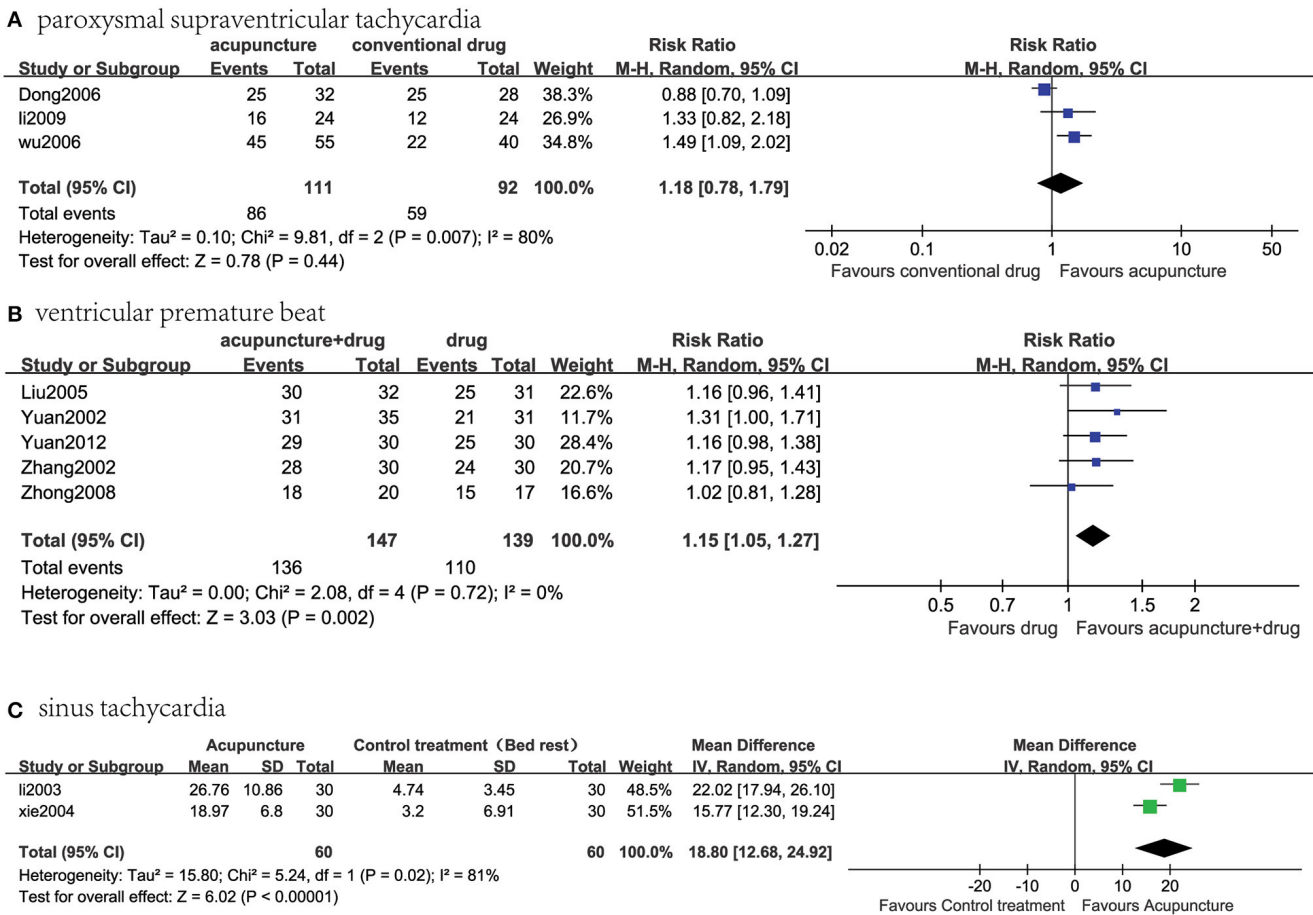
Meta-analysis of the effectiveness of acupuncture treatment compared with existing therapy for several kinds of arrhythmias. **(A)** Acupuncture vs. conventional drug therapy for PSVT; **(B)** acupuncture therapy plus the oral administration of AAD vs. the oral administration of AAD alone for VPB; **(C)** acupuncture vs. control treatment (neither acupuncture nor any other anti-arrhythmic treatment) for ST.

In total, 13 trials with 797 patients were available for analysis (Figure 1, Table 1; Yuan and Ai, 2002; Zhang and Xu, 2002; Li, 2003; Xie et al., 2004; Liu, 2005; Dong, 2006; Wu and Lin, 2006; Xu and Zhang, 2007; Zhong, 2008; Li and Guo, 2009; Lomuscio et al., 2011; Yuan and Zhao, 2012; Wang and Zhang, 2013). Three studies evaluated the effectiveness of acupuncture for PSVT (Figure 2A). Five studies assessed the effectiveness of acupuncture plus the oral administration of AAD compared with the oral administration of AAD only for the treatment of VPB (Figure 2B). Two studies tested the effects of acupuncture on Af compared with amiodarone. Two studies assessed the effect of a single acupuncture treatment in patients with ST; both studies showed significant effects on heart rate reduction. The last RCT investigated the effects of acupuncture on patients with premature beat (PB), and the results showed significant effects of acupuncture on reducing the number of PBs. Adverse events (AEs) were reported only in XU's study (Xu and Zhang, 2007), and no AEs were noted in the acupuncture group (Xu and Zhang, 2007).

**Table 1:** Summary of the included studies on acupuncture for cardiac arrhythmia.

**References**	**Design, condition (primary disease)**	**Randomization**	**Treatment course (day)**	**Age (year)**	**Intervention**		**Primary outcome**	**Result**
					**Treatment group**	**Control group**		
Li, 2003	RCT, ST	Referring to a random number table	1	19–65	AT(*n* = 30) (Linggui Bafa)	Bed-rest for 30 min(*n* = 30)	Response rate (Reduction of HR)	*P* < 0.05
Xie et al., 2004	RCT, ST	Referring to a random number table	1	18–63	AT(*n* = 30) (Linggui Bafa)	Bed-rest for 30 min(*n* = 30)	Response rate (Reduction of HR)	*P* < 0.05
Dong, 2006	RCT, PSVT	Unclear	1	26–62	AT(*n* = 32) (Neiguan)	Drug (Propafenone I.V.,*n* = 28)	Response rate (Reduction of HR)	*P* > 0.05
Wu and Lin, 2006	Quasi-RCT, PSVT	Referring to clinic record number	25	20–73	AT(*n* = 50) (wrist-ankle acupuncture)	Drug (Diltiazem P.O., *n* = 45)	Response rate (no more recurrent attack and restoration to NSR)	*P* < 0.05
Li and Guo, 2009	PSVT	Unclear	30	35–65	AT(*n* = 24) (Neiguan)	Drug (Diltiazem P.O., *n* = 24)	Response rate (no more recurrent attack and restoration to NSR)	*P* < 0.05
Xu and Zhang, 2007	RCT, Paroxysmal Af or AF	Referring to a random number table	1	48–69	AT(*n* = 40) (Neiguan, Danzhong, Qihai, Zhongwan, Zusanli, Xuehai, Fenglong)	Drug (Amiodarone I.V., *n* = 40)	(1) Response rate (restoration to NSR) (2) Average time of restoration to NSR	*P* < 0.01
Lomuscio et al., 2011	RCT, Persistent Af after EC	Unclear	70	59–67	AT(*n* = 17) (Neiguan, Shenmen, Xinshu)	Drug (Amiodarone I.V., *n* = 26)	Af recurrence rate	*P* > 0.05
Yuan and Ai, 2002	RCT, VPB	Unclear	30	40–65	AT(Neiguan, Shenmen, Zusanli, Huatuojiaji T4, T5)+Drug(Mexiletine P.O.), (*n* = 35)	Drug(Mexiletine P.O., *n* = 31)	(1) Response rate (% reduction of VBP on ECG > 50%) (2) Change of VPB-related symptom score	*P* < 0.05
Zhang and Xu, 2002	RCT, FVPB	Unclear	14	18–72	AT(Neiguan)+Drug(Mexiletine P.O.) (*n* = 30)	Drug(Mexiletine P.O., *n* = 30)	Response rate (symptom reduction and premature beat reduction <5 BPM)	*P* < 0.01
Liu, 2005	RCT, VPB	Unclear	14	17–76	AT(Neiguan, Shenmen)+Drug(Propafenone P.O.) (*n* = 32)	Drug (Propafenone P.O., *n* = 31)	Response rate (symptom reduction and HR reduction <5 BPM)	*P* < 0.05
Zhong, 2008	Quasi-RCT, VPB	Referring to clinic record number	42	17–61	AT(Neiguan, Shenmen, Xinshu, Juque)+Drug(Propafenone P.O.) (*n* = 20)	Drug (Propafenone P.O., *n* = 17)	Response rate (% reduction of VPB on ECG > 50%)	*P* > 0.05
Yuan and Ai, 2002	RCT, FVPB	Unclear	14	38–65	AT(Neiguan)+Drug(Wenxin granule P.O.) (*n* = 30)	Drug (Wenxin granule P.O., *n* = 30)	Response rate (% reduction of VPB on ECG > 50%)	*P* < 0.05
Wang and Zhang, 2013	RCT, PB	Unclear	12	42–70	AT(*n* = 34) (Lingtai, Shendao)	Sham AT (*n* = 31)	Response rate (% reduction of PB on ECG > 50%)	*P* < 0.05

*AT, acupuncture; RCT, randomized controlled trial; Quasi-RCT, quasi randomized controlled trial; Linggui Bafa, method for picking eight points linked with extraordinary meridians according to time; including acupuncture on Gongsun, Lieque, Shenmai, Zhaohai, Zulinqi, Houxi, Neiguan, and Waiguan, I.V, intravenous injection; PO, per os; NSR, normal sinus rhythm; HR, heart rate; BPM, beat per min; PSVT, paroxysmal supraventricular tachycardia; VPB, ventricular premature beat; FVPB, frequent ventricular premature beat; PB, premature beat; Af, atrial fibrillation; AF, atrial flutter; ST, sinus tachycardia; EC, electro-cardioversion; ECG, electrocardiogram*.

### Acupuncture vs. conventional drug therapy for paroxysmal supraventricular tachycardia

The results of the meta-analysis showed no statistically significant difference in the effectiveness of acupuncture therapy compared with conventional drug therapy for PSVT [*n* = 203; relative ratio (RR), 1.18, 95% confidence interval (CI) 0.78–1.79; *I*^2^ = 80%; *P* = 0.44; Figure 2A].

### Acupuncture + drug vs. conventional drug therapy for ventricular premature beat (VPB)

The results of the meta-analysis of these five RCTs showed a statistically significant benefit of acupuncture plus the oral administration of AAD on the response rate compared with the oral administration of AAD alone for VPB (*n* = 286; RR, 1.15, 95% CI 1.05–1.27; *I*^2^ = 0%; *P* = 0.002, Figure 2B).

### Acupuncture vs. control treatment (neither acupuncture nor any other anti-arrhythmic treatment) for sinus tachycardia

The results of the meta-analysis of these two RCTs showed a statistically significant benefit of acupuncture on the response rate compared with the control treatment (neither acupuncture nor any other anti-arrhythmic treatment) for ST (*n* = 120; MD, 18.80, 95% CI 12.68–24.92; *I*^2^ = 81%; *P* < 0.00001, Figure 2C).

### The risk of bias

**Figure 3:**
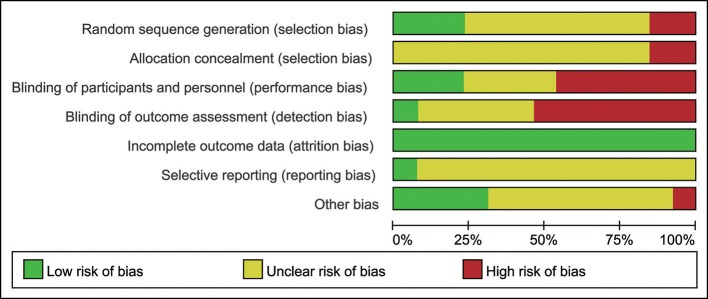
The risk of bias. A review of the authors' judgments about each risk of bias items are presented as percentages across all of the included studies. The quality of the selected studies was assessed according to the Cochrane criteria.

**Figure 4:**
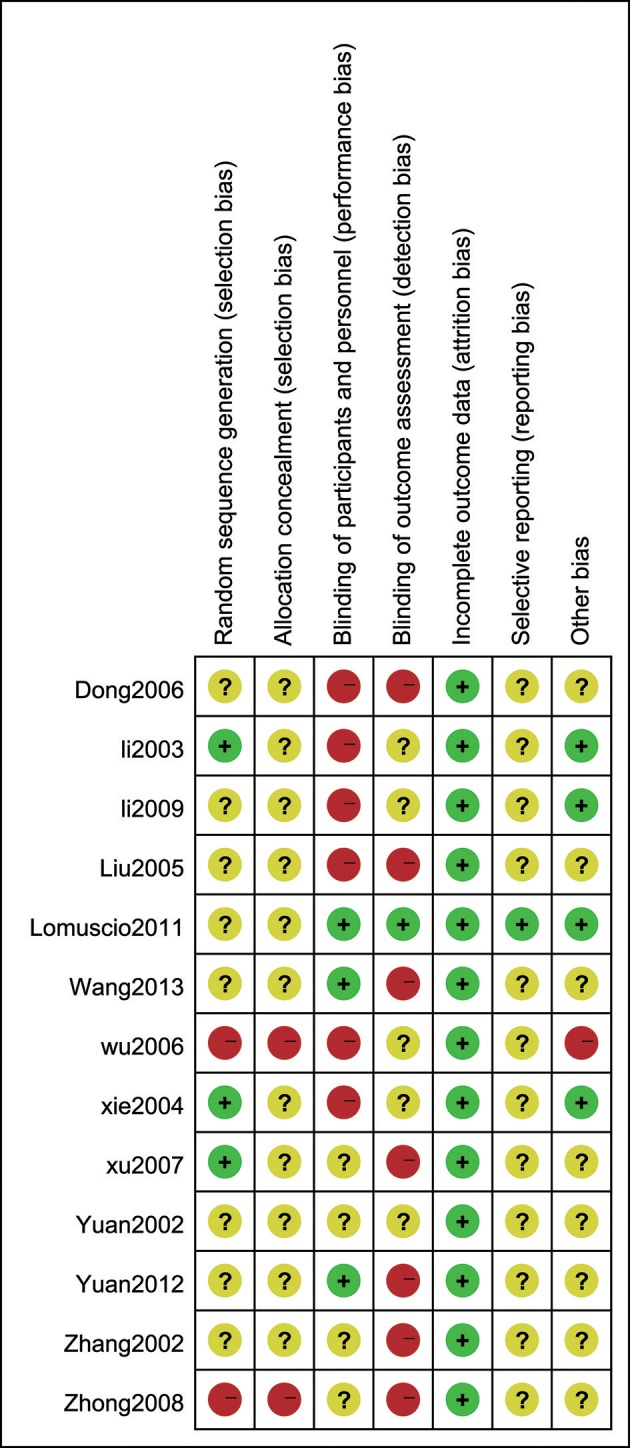
The risk of bias summary. A review of the authors' judgments about the risk of bias is included in each study.

A review of the authors' judgments about each risk of bias item presented as percentages across all of the included studies. The quality of the selected studies was assessed according to the Cochrane criteria (Figures 3, 4; Higgins and Altman, 2008).

### Publication bias

**Figure 5:**
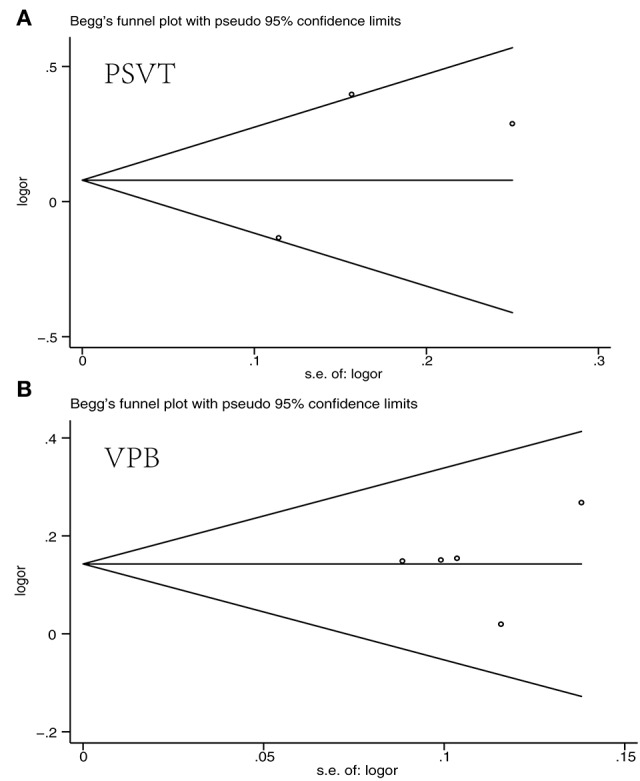
Funnel plot for publication bias [**(A)** PSVT; **(B)** VPB].

Funnel plot and Egger's test of publication bias were made for each analysis. However, as the number of included studies was small, the result might not be completely accuracy (Figure 5, Table 2).

**Table 2:** Egger's test of publication bias for PSVT and VPB.

**Arrhythmia**	**Std. Eff**.	**Coef**.	**Std. Err**.	**t**	***P* > *t***	**[95% CI]**
PSVT	slope	−0.486075	0.6192631	−0.78	0.576	−8.354558 7.382408
	bias	3.942069	4.131573	0.95	0.515	−48.55455 56.43869
VPB	slope	0.0427075	0.2579683	0.17	0.879	−0.7782627 0.8636777
	bias	0.9591691	2.44781	0.39	0.721	−6.830856 8.749194

*Coef, coefficient; Std. Eff, Standard Effect; Std. Err, Standard Error*.

### AEs on acupuncture

Adverse events (AEs) were reported only in XU's study (Xu and Zhang, 2007), and no AEs were noted in the acupuncture group (Xu and Zhang, 2007). In the amiodarone group, 1 patient developed hypotension, and 2 patients suffered vomiting. After symptomatic treatment, they didn't quit from the trial.

## Discussion

The main findings of this review were that the clinical evidence of acupuncture for cardiac arrhythmia is inconclusive, but in several groups of the meta-analysis (Figures 2B,C), acupuncture or acupuncture plus oral administration of AAD was found to be beneficial. Because of the poor methodological quality of the included studies, the evidence for acupuncture remains weak. Although there is not sufficient available data to draw a definite conclusion regarding the efficacy of acupuncture for arrhythmia, some evidences of the effect of acupuncture for the treatment of arrhythmia is provided (Figures 2B,C). Twelve trials used the response rate as an outcome measure and one trial used the recurrence rate as an outcome measure. The main source of heterogeneity may arise from several aspects. The most important reason is the number of invalid articles with small sample sizes. Other possible reasons include the variation in the acupuncture points chosen by doctors, the lack of a standard course of treatment, and that treatment duration is decided by the doctors and can be quite different. Therefore, heterogeneity arises from both clinical and methodological sources.

A total of 10 of the 13 studies used conventional acupuncture for arrhythmia treatment, primarily choosing the Neiguan (PC-6), Shenmen (HT-7), and Xinshu (BL-15) points. Two studies chose the Linggui Bafa method, a method for picking eight points linked with extraordinary meridians according to time. These acupuncture points include Gongsun, Lieque, Shenmai, Zhaohai, Zulinqi, Houxi, Neiguan, and Waiguan. This is an important part of time acupuncture therapy (Jihong, 2008). Another study used wrist-ankle acupuncture, a type of subcutaneous needling that is shallow and retained in the skin, which adjusts the nerves on multiple levels to balance or restore normal nerve function (Shu et al., 2011).

Most studies chose the Neiguan point (PC-6) to treat arrhythmia. Neiguan is the contact point of the pericardium meridian and is one of the eight confluent acupoints connecting the eight extra channels. This point is located in the portion of the Meridian of the Heart Minister situated in the forearm, along the course between the two tendons. The pericardium meridian controls blood flow and the pulse rate, and its malfunction is associated with anxiety, restlessness, and cardiac pain. The application of acupunctural therapy in the Neiguan spot is well-known in the western world for the treatment of chest pain, sickness, and vomiting during transcatheter arterial chemoembolization procedures (Shaoxiang, 1993). Experimental data also suggest that stimulation of the Neiguan point has been used to counteract the sensation of fullness or tension in the chest and palpitations and may determine a reduction in the electrocardiographic signs of myocardial ischemia and a reduction in the plasma endothelin levels (Hongwu, 1998). Furthermore, it has been reported that bilateral acupuncturing of Neiguan spots may affect the firing rate of the amygdala, exerting a modulatory function on the autonomic nervous system (Zhongfang, 1991). This suggests that acupuncture may exert its antiarrhythmic effect by acting on the autonomic nervous system.

The arrhythmia detection method was ECG in 6 studies, 24 h holter in 4 studies and ECG Monitoring System in 3 studies. It should be mentioned that in the study of Lomuscio, Patients' follow-up was planned with weekly phone contacts to investigate patient's symptoms in the first 4 weeks after cardioversion and monthly ambulatory visit with ECG recording thereafter for 12-month follow-up. Besides, for economical reasons they were unable to use a transtelephonic monitoring system to detect also asymptomatic or brief self-terminating Af episode.

PSVT is a reentry tachycardia related to the atrioventricular junction area and is commonly treated with propafenone and diltiazem. There were three articles about PSVT, in which acupuncture treatment was shown to affect PSVT; however, there was insufficient evidence to support its advantage in terms of the response rate compared with conventional drugs (Figure 2A).

VPB is a premature beat caused by ectopic excitation originating from the end of bush's bundle. There were five articles about VPB. The results of the meta-analysis showed a statistically significant benefit of acupuncture plus the oral administration of AAD, supporting a combination of acupuncture and medication for the treatment of VPB to achieve greater benefits compared with the oral administration of AAD alone for VBP (Figure 2B). However, it should be mentioned that the antiarrhythmic drugs used in these RCTs are inconsistent, Liu (2005) and Zhong (2008) used Propafenone, Zhang (Zhang and Xu, 2002) and Yuan (Yuan and Ai, 2002) used Mexiletine, while Yuan (Yuan and Zhao, 2012) used wenxin granule, this may cause heterogeneity.

Af is the most common arrhythmia in the world. Af is associated with obvious morbidity and mortality, including a four-to-five fold increase in the risk of stroke, (Wolf et al., 1978; Krahn et al., 1995) a doubling of the risk for dementia, (Ott et al., 1997; Miyasaka et al., 2007) a tripling of the risk for heart failure and a 40–90% increased risk of overall mortality (Krahn et al., 1995; Benjamin et al., 1998). The diminished quality of life and high health care costs associated with Af have spurred numerous investigations to develop more effective treatments for Af and its complications. Amiodarone is commonly used to treat Af and maintain sinus rhythm, but it can cause pulmonary fibrosis and other side effects. Two articles assessed the effectiveness of acupuncture compared with Amiodarone for Af. Unfortunately, these 2 studies were quite different in the primary outcome measures, it was inappropriate to combine the data to reach a further conclusion. In Xu's study, 80 patients with paroxysmal atrial fibrillation were randomly divided into acupuncture treatment group and amiodarone treatment group, the sinus rhythm restoration rate and the time of cardioversion to sinus rhythm were set as the primary outcomes, and the result showed that the sinus rhythm restoration rate in acupuncture treatment group was significantly higher than in amiodarone treatment group (*P* < 0.01). Xu also compared the time of cardioversion to sinus rhythm in these 2 groups, in acupuncture treatment group the average conversion time was found to be significantly shorter (*P* < 0.01; Xu and Zhang, 2007). In Alberto Lomuscio's study, 80 patients with persistent Af after restoring sinus rhythm with electrical cardioversion were observed, patients who were already on amiodarone treatment constituted the amiodarone reference group, others were randomly allocated to receive acupuncture, sham acupuncture, or neither acupuncture nor antiarrhythmic therapy. Patients in the acupuncture and sham acupuncture groups attended 10 acupuncture sessions on a once-a-week basis. During a 12-month follow-up, Af recurred in 35 patients. Cumulative Af recurrence rates in the amiodarone, acupuncture, sham acupuncture, and control patients were 27, 35, 69, and 54%, respectively (*P* = 0.0075, log-rank test). Compared with AMIO group, recurrence rate was similar in acupuncture patients (*P* = 0.801) but significantly higher in sham acupuncture and control patients (*P* = 0.009 and *P* = 0.017, respectively) after adjustment for ejection fraction, hypertension, and left atrial diameter using Cox modeling (Lomuscio et al., 2011). Several clinical and experimental reports have indicated that autonomic nervous system may favor the initiation and maintenance of Af episodes, it is therefore possible to speculate that the antiarrhythmic action of acupuncture is through modulation of the sympathetic and parasympathetic nervous system, a possibility that is in need of further study (Andrew and Mehmet, 2014).

ST is one of the most common arrhythmias in clinical practice, but it is very easily neglected. At present, ST is an important factor affecting the prognosis of some types of heart disease, such as heart failure. Two articles assessed the use of acupuncture and a control treatment (neither acupuncture nor any other anti-arrhythmic treatment) for ST in this meta-analysis. The results of the meta-analysis showed a statistically significant benefit of acupuncture, suggesting its advantage in the response rate compared with the control group (Figure 2C). In this study, the results have a very high heterogeneity (*I*^2^ = 81%), which may be a result of several factors. First and foremost, there are only two studies included in this meta-analysis, which may lead to high heterogeneity. Second, patients were consistently treated by acupuncture at different acu-points. Third, the sample sizes in these two studies were small. Additionally, there were differences in the doctors' levels of expertise. Finally, variation may arise from differences in the diagnostic equipment between hospitals. Therefore, clinical and methodological sources may have influenced the heterogeneity of the statistics.

One limitation of this analysis is that the evidence associated with acupuncture treatment for cardiac arrhythmia is limited; most reports in the literature of acupuncture treatment for this disease are of low quality. Many of these studies have problems, such as a small sample size, unclear randomization, and a lack of long-term curative effect data. Only one study conducted follow-up for 1 year, (Lomuscio et al., 2011) and only three studies explicitly mentioned blinding (Lomuscio et al., 2011; Yuan and Zhao, 2012; Wang and Zhang, 2013). Manipulation, the retaining time, depth, and needle direction can all cause heterogeneity in clinical trials of acupuncture. The results of these clinical studies are still controversial. However, this review demonstrates the current level of evidence supporting acupuncture treatment for cardiac arrhythmia treatment and offers basic knowledge of acupuncture interventions for future clinical trials.

In summary, our meta-analysis demonstrates that clinical efficacy of AAD is not better than acupuncture. Furthermore, in sub-group analysis, acupuncture with or without AAD, shows a clear benefit in treating VPB and ST. However, RCTs with methodological rigor should still be conducted, in addition to the adoption of validated outcomes trials. We should continue to expand the cumulative meta-analysis of future trials, especially RCTs, and find safe and well-tolerated interventions to help arrhythmia patients gain treatment benefits.

## Author contributions

DH and YX design the project. DH, YX, YL, HB, and BL coordinated and collected the clinical evaluations. YX, DH, YL, HB, BL, YG, and HS organized and summarized the data. YL, BL, YX, DH, and HB analyzed the data and made the figures and table. YX, DH, YL, HB, and BL wrote the manuscript. All co-authors contributed to editing and proving of manuscript.

### Conflict of interest statement

The authors declare that the research was conducted in the absence of any commercial or financial relationships that could be construed as a potential conflict of interest.

## Abbreviations

AAD: anti-arrhythmic drug
AE: Adverse event
Af: atrial fibrillation
AT: acupuncture
BPM: beat per min
EC: electro-cardioversion
ECG: electrocardiogram
FVPB: frequent ventricular premature beat
HR: heart rate
I.V: intravenous injection
NSR: normal sinus rhythm
PB: premature beat
P.O: per os
PSVT: paroxysmal supraventricular tachycardia
Quasi-RCT: quasi randomized controlled trial
RCT: randomized controlled trial
ST: sinus tachycardia
VPB: ventricular premature beat.

## References

[B1] Andrew B. Mehmet K. (2014). Review of complementary and alternative medical treatment of Arrhythmias. Am. J. Cardiol. 113, 897–903. 10.1016/j.amjcard.2013.11.044 24528618

[B2] Benjamin E. J. Wolf P. A. D'Agostino R. B. Silbershatz H. Kannel W. B. Levy D. (1998). Impact of atrial fibrillation on the risk of death: the Framingham Heart Study. Circulation 98, 946–952. 10.1161/01.CIR.98.10.946 9737513

[B3] Burashnikov A. Antzelevitch C. Barajas-Martinez H. Antzelevitch C. (2012). Atrial-selective inhibition of sodium-channel current by Wenxin Keli is effective in suppressing atrial fibrillation. Heart Rhythm 9, 125–131. 10.1016/j.hrthm.2011.08.027 21884675 PMC3236802

[B4] Camm A. J. (2017). Hopes and disappointments with antiarrhythmic drugs. Int. J. Cardiol. 237, 71–74. 10.1016/j.ijcard.2017.03.056 28365182

[B5] Chen Y. Shaoping N. Hai G. Tao S. Xiaoqiu L. Fei T.. (2013). The effects of Wenxin Keli on P-wave dispersion and maintenance of sinus rhythm in patients with paroxysmal atrial fibrillation: a meta-analysis of randomized controlled trials. Evid. Based Complement. Altern. Med. 2013:245958. 10.1155/2013/245958 24368925 PMC3867920

[B6] Dong S. J. (2006). 32 cases of paroxysmal supraventricular tachycardia in acupuncture Neiguan. J. Henan. Univ. Chin. Med. 21, 69–70. 10.16368/j.issn.1674-8999.2006.06.051

[B7] Han B. J. Wang F. (2008). Acupuncture treatment for 98 cases of ventricular premature beat. J. Tradit. Chin. Med. 28, 86–89. Available online at: http://kns.cnki.net/KCMS/detail/detail.aspx?dbcode=CJFQ&dbname=CJFD2008&filename=ZYYW200802002&v=MDE0OTZXNy9PUHpUU2ViRzRIdG5Nclk5RlpvUjhlWDFMdXhZUzdEaDFUM3FUcldNMUZyQ1VSTDJmWXVSdEZDbms= 18652111 10.1016/s0254-6272(08)60021-7

[B8] Higgins J. Altman D. (2008). Chapter 8: assessing risk of bias in included studies, in Cochrane Handbook for Systematic Reviews of Interventions, eds Higgins J. Green S. (Chichester: John Wiley & Sons), 187–242.

[B9] Hongwu L. (1998). Specific therapeutic effect of Neiguan on heart disease. Int. J. Clin. Acupunct. 9, 303–305.

[B10] Jihong W. (2008). The theoretical origin and clinical application of the eight methods of Linggui Bafa. J. New Chin. Med. 40, 111–112. 10.13457/j.cnki.jncm.2008.02.051

[B11] Kim T. H. Choi T. Y. Lee M. S. Ernst E. (2011). Acupuncture treatment for cardiac arrhythmias: a systematic review of randomizedcontrolled trials. Int. J. Cardiol. 49, 263–265. 10.1016/j.ijcard.2011.02.049 21421272

[B12] Krahn A. D. Manfreda J. Tate R. B. Mathewson F. A. Cuddy T. E. (1995). The natural history of atrial fibrillation: incidence, risk factors, and prognosis in the Manitoba follow-up study. Am. J. Med. 98, 476–484. 10.1016/S0002-9343(99)80348-9 7733127

[B13] Li H. (2003). The transient effect of acupoint selection of Linggui Bafa on sinus tachy cardia. Chin. Acupunct. Moxibusion 23, 132–134. Available online at: http://kns.cnki.net/KCMS/detail/detail.aspx?dbcode=CJFQ&dbname=CJFD2003&filename=GSZB200304002&v=MDY0OTJxNDlGWm9SOGVYMUx1eFlTN0RoMVQzcVRyV00xRnJDVVJMMmZZdVJ0RkNubVY3cklJajdSYkxHNEh0TE0=

[B14] Li Y. X. Guo R. S. (2009). Clinical observation on acupuncture on Neiguan for paroxysmal supraventricular tachycardia. Heilongjiang Med. J. 22, 699 10.14035/j.cnki.hljyy.2009.05.013

[B15] Lin Q. (2015). Present situation and prospect of traditional Chinese medicine on the treatment of Arrhythmia. Chin. J. Integr. Med. Cardiol. Cerebrovasc. Dis. 2, 129–131. Available online at: http://kns.cnki.net/KCMS/detail/detail.aspx?dbcode=CJFQ&dbname=CJFDLAST2015&filename=ZYYY201502001&v=MjQ1MDV4WVM3RGgxVDNxVHJXTTFGckNVUkwyZll1UnRGQ25uVUx6SlB6VFNkN0c0SDlUTXJZOUZaWVI4ZVgxTHU=

[B16] Liu L. Y. (2005). Clinical trial of integrated traditional and western medicine for frequent ventricular premature beat. J. Emerg. Tradit. Chin. Med. 14, 619–621. Available online at: http://kns.cnki.net/KCMS/detail/detail.aspx?dbcode=CJFQ&dbname=CJFD2005&filename=ZYJZ200507015&v=MTUyMTMzcVRyV00xRnJDVVJMMmZZdVJ0RkNublY3M0pQelRCZExHNEh0VE1xSTlFWVlSOGVYMUx1eFlTN0RoMVQ=

[B17] Lomuscio A. Belletti S. Battezzati P. M. Lombardi F. (2011). Efficacy of acupuncture in preventing atrial fibrillation recurrences after electrical cardioversion. J. Cardiovasc. Electrophysiol. 22, 241–247. 10.1111/j.1540-8167.2010.01878.x 20807278

[B18] Miyasaka Y. Barnes M. E. Petersen R. C. Cha S. S. Bailey K. R. Gersh B. J.. (2007). Risk of dementia in stroke-free patients diagnosed with atrial fibrillation: data from a community-based cohort. Eur. Heart J. 28, 1962–1967. 10.1093/eurheartj/ehm012 17459900

[B19] Mozaffarian D. Benjamin E. J. Go A. S. Arnett D. K. Blaha M. J. Cushman M.. (2016). Heart disease and stroke statistics-2016 update: a report from the American Heart Association. Circulation 133, e38–e360. 10.1161/CIR.0000000000000350 26673558

[B20] Ott A. Breteler M. M. de Bruyne M. C. van Harskamp F. Grobbee D. E. Hofman A. (1997). Atrial fibrillation and dementia in a population-based study. Stroke 28, 316–321. 10.1161/01.STR.28.2.316 9040682

[B21] Shaoxiang L. (1993). Magnetic disk applied on neiguan point for prevention and treatment of cisplatin-induced nausea and vomiting. J. Tradit. Chin. Med. 11, 181–183. 1749262

[B22] Shu S. Li T. M. Fang F. F. He H. L. Zhou Q. H. Gu W.. (2011). Relieving pre-exam anxiety syndrome with wrist-ankle acupuncture: a randomized controlled trial. J. Chin. Integr. Med. 9, 605–610. 10.3736/jcim20110605 21669163

[B23] Wang R. Zhang N. N. (2013). Impacts of electroacupuncture at Lingtai (GV10) and Shendao(GV11) on premature heartbeat. Chin. Acupunct. Moxibusion 33, 385–387. 10.13703/j.0255-2930.2013.05.004 23885605

[B24] Wolf P. A. Dawber T. R. Thomas H. E. Jr. Kannel W. B. (1978). Epidemiologic assessment of chronic atrial fibrillation and risk of stroke: the Framingham study. Neurology 28, 973–977. 10.1212/WNL.28.10.973 570666

[B25] Wu R. D. Lin L. F. (2006). Clinical observation on wrist-ankle acupuncture for treatment of paroxysmal supraventricular tachycardia. Zhongguo Zhen Jiu 26, 854–856. 10.13703/j.0255-2930.2006.12.006 17313005

[B26] Xie H. Li H. Zhao C. (2004). Effect of Linggui Bafa acupuncture on heart rate in the patient of sinus tachycardia. Chin. Acupunct. Moxibust. 24, 449–451. 10.13703/j.0255-2930.2004.07.003

[B27] Xu H. K. Zhang Y. F. (2007). Comparison between therapeutic effects of acupuncture and intravenous injection of amiodarone in the treatment of paroxymal atrial fibrillation and atrial flutter. Zhongguo Zhen Jiu 27, 96–98. 10.13703/j.0255-2930.2007.02.008 17370488

[B28] Yuan X. X. Zhao L. G. (2012). Clinical observation on the efficacy of combined acupuncture and Wenxin granule in treating ventricular premature beat medical innovation of China. Med. Innov. China 9, 138–139. Available online at: http://kns.cnki.net/KCMS/detail/detail.aspx?dbcode=CJFQ&dbname=CJFD2012&filename=ZYCX201201093&v=MDE2MDVMMmZZdVJ0RkNqa1dyL05QelRJZHJHNEg5UE1ybzlNWjRSOGVYMUx1eFlTN0RoMVQzcVRyV00xRnJDVVI=

[B29] Yuan Z. J. Ai B. W. (2002). Clinical trial of acupuncture plus western medication for ventricular premature beat. Chin. J. Integr. Trad. West. Med. 22, 312–313. Available online at: http://kns.cnki.net/KCMS/detail/detail.aspx?dbcode=CJFQ&dbname=CJFD2002&filename=ZZXJ200204037&v=MzE4NjF6ZlRaTEc0SHRQTXE0OUdZNFI4ZVgxTHV4WVM3RGgxVDNxVHJXTTFGckNVUkwyZll1UnRGQ2prVTc3T1A=

[B30] Zhang J. Z. Xu W. H. (2002). Frequent ventricular extrasystole treated by needling neiguan (PC 6) plus oral administration of mexiletine—a report of 30 cases. New J. Tradit. Chin. Med. 34, 45–46. 10.13457/j.cnki.jncm.2002.11.021 15119171

[B31] Zhong C. (2008). Observation on the efficacy of combined acupuncture and medicine in treating ventricular premature beat organic heart disease. Shanghai J. Acu. Mox. 27, 15–16. 10.13460/j.issn.1005-0957.2008.05.007

[B32] Zhongfang L. (1991). Role of amygdaloid nucleus in the correlation between the heart and acupoint Neiguan in rabbits. J. Tradit. Chin. Med. 11, 128–138. 1861520

